# Une arthrite septique sur prothèse totale de genou à *Pasteurella multocida*: à propos d'un cas

**DOI:** 10.11604/pamj.2015.20.366.6155

**Published:** 2015-04-15

**Authors:** Biova Teko Kouevidjin, Jonathan Sylvanus Bassinga

**Affiliations:** 1 Service de Chirurgie Orthopédique et Traumatologique, CHU Ibn Sina, Rabat, Maroc

**Keywords:** PTG, Pasteurella multocida, Arthrite, Total knee prosthesis, Pasteurella multocida, arthritis

## Abstract

Une arthrite septique sur PTG est due essentiellement au Staphylococcus aureus suivie des staphylocoques à coagulase négative, et les streptocoques. Au cours de ses 40 dernières années très peu de cas d'infection sur arthroplastie à Pasteurella multocida ont été rapporté. La présentation clinique n'a rien de spécifique.la contamination survient après une morsure, griffure ou léchage d'un chat. L'interrogatoire et l'examen bactériologique est la clé du diagnostique. Nous rapportons le cas d'une patiente de 84 ans qui présente une infection a Pasteurella multocida suite à une morsure du chat 06 jours au paravent. Elle a bénéficié d'une prise en charge chirurgicale par lavage et synovectomie et une bi-antibiothérapie avec bonne évolution.

## Introduction

Les cocci gram positifs constituent la cause la plus fréquente d'infection de prothèse articulaire. L'infection d'une prothèse à Pasteurella est une complication rare et s'agit souvent du *Pasteurella multocida*. Elle est inoculée à l'homme par des morsures ou griffures de chat. Nous rapportons ici une infection de prothèse totale de genou (PTG) par Pasteurella multocida.

## Patient et observation

**Figure 1:**
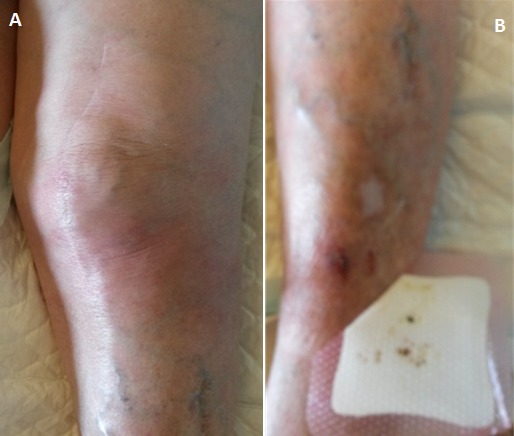
a)genou gauche infecté avec rougeur et ancienne cicatrice opératoire; b) traces de griffure de chat au niveau de la crête tibiale

**Figure 2:**
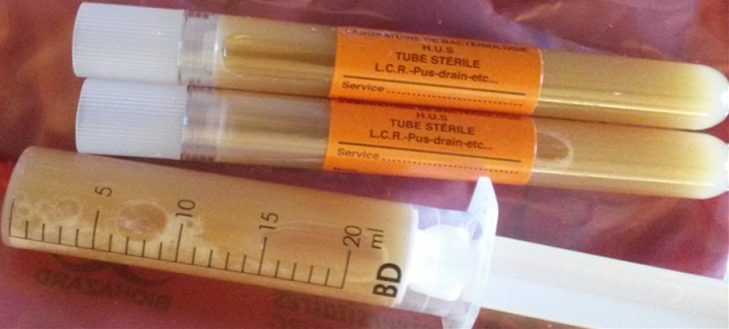
Ponction articulaire préopératoire

Il s'agit d'une patiente de 84 ans ayant comme antécédents une PTG bilatérale en 2012, un diabète insulinorequérant, une obésité morbide, une HTA et une embolie pulmonaire suite à une TVP. L'intervention n’était compliquée d'aucun problème mécanique ou infectieux. En juin 2014 elle a été victime d'une morsure de chat au niveau de sa jambe gauche. Elle a consulté son médecin traitant 48h après suite à une rougeur et douleur légère au niveau de la jambe. La patiente présentait une fébricule à 38.1 et une rougeur, ce qui a conduit son médecin traitant à la mettre sous Acide fucidique. Trois jours plus tard elle est victime d'une chute banale ayant occasionnée une fracture articulaire du radius distal gauche. A son admission à l'hôpital outre le diagnostic de sa fracture du radius d'indication chirurgicale, la patiente présentait un syndrome infectieux avec une fièvre à 38.8 avec une CRP à 260.2 mg/l; 11250 de leucocytes avec 9860 de Polynucleaire Neutrophile. Transférée dans un service de médecine interne pour recherche étiologique, la patiente est mise sous Rocéphine 2g/j et Gentamycine. C’était lors d'une reprise interrogatoire au lendemain de son hospitalisation soit 06 jours après la morsure du chat qu'elle rapporte une douleur modérée au niveau de son genou gauche. Sur le plan clinique elle présente une douleur à la palpation et à la mobilisation avec un épanchement modéré et une rougeur et chaleur. Un flexum de 10°. Une petite rougeur au niveau de la face antéro-externe de la jambe laissant apparaitre 4 traces de griffure (Figure 1). Un examen radiologique n'objective pas de signe de descellement de prothèse. Une ponction articulaire faire avec une asepsie rigoureuse donne un liquide trouble (Figure 2) (Tableau 1). Le lendemain nous avons réalisé un lavage avec synovectomie (Tableau 2) et changement du Polyéthylène ainsi qu'une ostéosynthèse du radius distal. L'intervention s'est déroulée sans problème et les suites opératoires étaient simples. Les analyses cytobactériologiques de la ponction articulaire et du prélèvement synovial ont objectivé la présence du ***Pasteurella multocida multi sensible***. La patiente est sortie sous Ofloxacine 200mg chaque 12h et Amoxicilline 1g chaque 8h pendant 02 mois.LA CRP à la sortie au 6eme jour postopératoire était de 82.7. Au dernier recule à 06 mois elle présente un genou indolore, fonctionnel sans flexum et une CRP ≤ 6.

**Tableau 1:** Examen cytologique de la ponction articulaire préopératoire

Origine prélèvement	Unité de mesure	Liquide articulaire
Aspect	-	Purulent
Cristaux	-	Rares cristaux
Ragocytes	-	Absence
Hématies	10^6^/l	1200
Cel nucléées	10^6^/l	100800
Cytologie	Cell	100
P neutro	-	94,5
Acide urique	µmol/l	286
Protéines	g/l	52.8

**Tableau 2:** Examen cytobactériologique du liquide articulaire et biopsie synoviale postopératoire

Echantillon	Fragment synovial	
Leucocytes mononuclées	Rares	
Cocci Gram(+) Examen direct	Rares	
Culture	Pasteurella multocida (souche non productrice de beta-lactamase)	
Antibiogramme	Penicilline G	Sensible
	Amoxicilline	Sensible
	Amoxicilline-Acide clavulanique	Sensible
	Ticarcilline- Acide clavulanique	Sensible
	Piperacilline	Sensible
	Piperacilline-Tazobactam	Sensible
	Cefalotine	Sensible
	Cefotaxime	Sensible
	Cefoxitine	Sensible
	Ceftazidime	Sensible
	Imipeneme	Sensible
	Gentamicine	Résistant
	Tobramycine	Résistant
	Ofloxacine	Sensible
	Ciprofloxacine	Sensible
	Doxycycline	Sensible

## Discussion

Les Pasteurella, d'origines canines, sont des germes bacilles Gram négatifs responsables d'infection humaine locale ou systémique. La majorité des infections à Pasteurella font suites à un contact avec un chat. L'inoculation se fait par morsure ou griffure de l'animal mais aussi par un simple léchage des plaies et dans 3% des cas, il n’était pas montré de liens évidents avec un animal [1]. Les infections humaines à Pasteurella peuvent être à l'origine d'arthrites septiques soit par inoculation directe du germe, soit par voie hématogène. Des patients présentant généralement un terrain d'immunodepression. Vu la situation plus superficielle des PTG, elles sont plus exposées que les autres prothèses. Le tableau clinique initial est le plus souvent une arthrite aiguë, avec apparition des symptômes de 24 heures à trois mois. Les prothèses infectées par Pasteurella posent plusieurs problèmes diagnostiques car sur le plan clinique, les symptômes sont souvent atténués par les immunosuppresseurs [2]. La première description d'une infection sur PTG à *Pasteurella multocida* remonte en 1973 par ARVAN et al [3]. En 2006 Camelia et Marculescu [4] rapportent 19 infections sur prothèses par P. multocida. Un seul cas d'infection sur PTG à Pasteurella Canis est décrit dans la littérature [5]. Il n'existe pas de spécificité en termes de prise en charge. Une arthrite aigüe diagnoctiquée très tôt sans signe de descellement peut bénéficier d'un simple lavage et synovectomie comme dans notre cas. En cas de descellement le changement e un temps ou en deux temps est une affaire d’école. Les antibiotiques recommandés sont les bêta-lactamines ou les tétracyclines, mais certaines souches produisent des bêta-lactamases et il peut donc être intéressant d'y associer de l'acide clavulanique. La durée du traitement est de 06 semaines à 12 semaines. Dans notre cas après quelques jours d'antibiothérapie parentérale et dès réception de l'antibiogramme la patiente est mise sous Ofloxacine et Amoxicilline pendant 02 mois avec bonne évolution.

## Conclusion

Une infection à *Pasteurella multocida* est peu fréquente dans la littérature. Ce diagnostic est à évoquer systématiquement à chaque fois qu'il y a une notion de contacte canine dans l'histoire de la maladie Une antibiothérapie prophylactique par acide clavulanique après une morsure ou griffure de chat et de chien est recommandée en l'occurrence si le patient est immunodéprimé, ou porteur d'une arthroplastie.
